# Active Constituents from *Liriope platyphylla* Root against Cancer Growth *In Vitro*

**DOI:** 10.1155/2013/857929

**Published:** 2013-05-16

**Authors:** Hui-Chun Wang, Chin-Chung Wu, Ti-Sheng Cheng, Ching-Ying Kuo, Yu-Chi Tsai, Shang-Yu Chiang, Teng-Song Wong, Yang-Chang Wu, Fang-Rong Chang

**Affiliations:** ^1^Graduate Institute of Natural Products, College of Pharmacy, Kaohsiung Medical University, Kaohsiung 80708, Taiwan; ^2^Cancer Center, Kaohsiung Medical University Hospital, Kaohsiung 80756, Taiwan; ^3^School of Chinese Medicine, College of Chinese Medicine, China Medical University, Taichun 40402, Taiwan; ^4^Chinese Medicine Research and Development Center and Center for Molecular Medicine, China Medical University Hospital, Taichung 40447, Taiwan

## Abstract

*Liriope spicata* is a well-known herb in traditional Chinese medicine, and its root has been clinically demonstrated to be effective in the treatment of metabolic and neural disorders. The constituents isolated from *Liriope* have also recently been shown to possess anticancer activity, although the mechanism of which remains largely unknown. Here, we illustrate the anticancer activity of LPRP-9, one of the active fractions we fractionated from the *Liriope platyphylla* root part (LPRP) extract. Treatment with LPRP-9 significantly inhibited proliferation of cancer cell lines MCF-7 and Huh-7 and down-regulated the phosphorylation of AKT. LPRP-9 also activates the stress-activated MPAK, JNK, p38 pathways, the p53 cell-cycle checkpoint pathway, and a series of caspase cascades while downregulating expression of antiapoptotic factors Bcl-2, Bcl-XL, and survivin. Such activities strongly suggest a role for LPRP-9 in apoptosis and autophagy. We further purified and identified the compound (−)-Liriopein B from LPRP-9, which is capable of inhibiting AKT phosphorylation at low concentration. The overall result highlights the anticancer property of LPRP-9, suggests its mechanism for inhibition of proliferation and promotion of cell death for cancer cells via regulation of multitarget pathways, and denotes the importance of purifying components of fraction LPRP-9 to aid cancer therapy.

## 1. Introduction

“Radix Liriopis,” or the tuber of the *Liriopis* plant, is a medicinal ingredient traditionally used in several Asian countries. Based on ancient records, it has been utilized for hundreds of years to relieve symptoms of dry hacking coughs, dry tongue and mouth, insomnia, and constipation. Modern scientific studies have also confirmed that *Liriope* root extract reduces inflammation and alleviates symptoms of metabolic and neurodegenerative disorders, including diabetes and pathological obesity [1–4]. Many locals in southern Taiwan also believe that consumption of the tuber of *Liriope platyphylla* helps extend the life expectancy of cancer patients and, therefore, cultivate *Liriope platyphylla* in vast quantities. Constituents isolated from *Liriope* have recently been demonstrated to possess anticancer activity [5, 6], which forms our basis for examining the cellular and molecular impacts of fractions of *Liriope platyphylla* root extract on human cancer cell lines.

Many anticancer medicinal compounds isolated from plants in use today have the ability to interfere with important cellular functions, especially on signal transduction processes which confer selective advantages for progressive cancer cells. PI3 K/Akt pathway is one such process, which plays critical roles in mammalian cell growth and survival signaling. This pathway has been shown to be activated constitutively in a variety of cancers, making it a major focus in the development of anticancer agents. PI3 K/Akt signaling is activated downstream of receptor tyrosine kinases (RTKs) and causes phosphorylation of a wide range of other downstream targets that regulate cell growth, cell cycle progression, and cell migration. Forkhead box O (FoxO) transcription factors are among those targets and are known tumor suppressors that are negatively regulated by AKT phosphorylation. Phosphorylated FoxO is restrictively localized in the cytoplasm and unable to transactivate its target genes associated with apoptosis and cell cycle arrest, making PI3 K/Akt/FoxO pathway an attractive target for cancer prevention and treatment [7].

Besides PI3K/AKT/FoxO, the ERK mitogen-activated protein kinase (MAPK) pathway is another interesting pathway which consists of cytoplasmic signaling activated by growth receptor and is often highly activated in cancer cells, resulting in uncontrolled growth. ERK1/2 MAPK pathway is downstream of the oncogene RAS and has been utilized as a biomarker for EGFR activation. The EGFR/Ras/Raf/MEK/ERK signaling has been a hot area of research in identification of novel targets to assist in understanding of oncogenesis and discovery of new cancer treatment options [8]. The other two major components of MAPKs, JNK and p38, are activated by cell stress, inflammation, and growth factors. JNKs and p38 MAPKs signaling cascades can positively regulate cell cycle arrest and apoptosis in stressed cells [9]; and has been activated extensively in chemotherapy [10].

In this study, an active fraction of *Liriope platyphylla* root part extract (LPRP), named LPRP-9, was isolated using bioassay guided fractionation, and its anticancer activity was assayed. LPRP-9 was found to inhibit growth and induce apoptosis in cancer cell lines, and the extent of impact on the PI3K and MAPK pathways by LPRP-9 was further investigated. To identify the constituent that contributes to partial effects of LPRP-9, a component isolated from LPRP-9, (−)-Liriopein B, was further evaluated.

## 2. Materials and Methods

### 2.1. Cell Culture

Hep 3B (hepatocellular carcinoma; ATCC HB-8064, BCRC 60434), MDA-MB-231 (breast adenocarcinoma; ATCC HTB-26, BCRC 60425), MCF-7 (breast adenocarcinoma; ATCC HTB-22, BCRC 60436), and A549 (lung adenocarcinoma; ATCC CCL-185, BCRC 60074) human cell lines were purchased from Bioresource Collection and Research Center (BCRC, Hsinchu, Taiwan), and were authenticated by American Type Culture Collection (ATCC, Manassas, VA). Human hepatocellular carcinoma cells Huh-7 was provided by Dr. Chien-Chih Chiu. All cells were maintained in Dulbecco's modified Eagle's medium (DMEM, Sigma-Aldrich) except MDA-MB-231 cells which were maintained in RPMI-1640 media (Sigma-Aldrich) and supplemented with 10% fetal bovine serum (Gibco). All cells were maintained at 37°C in a 5% CO_2_ incubator (NuAire).

### 2.2. Drug Preparations and Treatments

LPRP, LPRP-9, and (−)-Liriopein B were purified by us, and their purity (99.5%) was identified by HPLC and NMR analysis [11], and like positive drugs doxorubicin (Sigma-Aldrich) and resveratrol (Tocris) were dissolved in DMSO and added to the culture medium 24 or 48 hours before the cells were harvested. For each treatment, cells were exposed to different drug concentrations while solvent DMSO concentration was adjusted and limited to 0.01% (v/v). A culture medium containing equivalent amounts of DMSO and no drugs served as negative control. Inhibitors Z-VAD-FMK, 3-methyladenine, and pifithrin-*α* were purchased from Tocris bioscience and added to culture medium 30 min before LPRP-9 or positive control drug treatment.

### 2.3. Cell Survival Assay

A stock solution of MTT (3-(4,5-dimethylthiazol-2-yl)-2,5-diphenyltetrazolium bromide; Sigma-Aldrich) was prepared in phosphate buffer saline (PBS) at a concentration of 5 mg/mL, filtered through a 0.22 *μ*m filter, and stored at 4°C in the dark. 1 × 10^4^ cells were seeded per well in 100 ul media on 96 well plates and treated 24 hours later with test drugs described previously. After 48 hours of drug incubation, cell growth media was replaced by complete media with MTT in a final concentration of 0.5 mg/mL and then incubated for 4 hours. After removal of the medium from the wells by aspiration, 100 *μ*L of DMSO were added to each well to dissolve the formazan crystals. Absorbance at 550 nm was determined using microplate photometer (Multiskan Ascent; Thermo Scientific). Percentage of survival was calculated using the following formula: cell survival (% of control) = OD_test_/OD_control_× 100%. Triplicate experiments were performed for each condition, and mean ± standard error (SE) was determined.

### 2.4. Chromosomal Fragmentation

The chromosomal fragmentation assay was used to determine the amount of chromosomes that is degraded upon treatment of cells with experiment drugs. Cells were exposure to doxorubicin or LPRP-9 for 24 hours, and harvested cells are then lysed by 400 *μ*L of lysis buffer (10 mM Tris, pH 8.0, 10 mM EDTA, 100 mM NaCl, and 1% SDS) supplemented with proteinase K at final concentration of 0.2 mg/mL. After incubation at 55°C water bath for 1 hour, the clear and well-dissolved cell lysate was partitioned with 400 *μ*L of phenol and chloroform. The aqueous fraction containing chromosomal DNA was separated from organic solvent using high-speed centrifugation, and ethanol precipitation assay was used to concentrate nucleic acids in a separate tube. Equivalent amounts of DNA samples were analyzed for degree of DNA fragmentation by 0.8% agarose gel electrophoresis in Tris-borate-EDTA buffer at a voltage of 100 V for 30 min. DNA was stained with florescent ethidium bromide, and images were captured using the Red Imaging System from Alpha Innotech.

### 2.5. Immunoblotting

Immunoblot assay was used to detect protein expression as described previously [12]. Primary antibodies were incubated in PBST containing 1% nonfat milk for 2 hours, and binding was detected by horseradish peroxidase-coupled secondary antibodies (Jackson ImmunoResearch Laboratories) followed by ECL detection (Millipore). The images of nonsaturated bands were captured using LAS-4000 mini luminescent image analyzer (Fujifilm). The primary antibodies against p38 *α*/*β*, LC3B, Bcl-2, Bcl-xL, Bax, Bad, Bid, AKT, caspase-3, phosphor- AKT (Thr308), AKT (Ser473), ERK (Thr202/Tyr204), p38(Thr180/Tyr182), and Fox03a (Ser318/321) were purchased from cell signaling; ERK, JNK, p53 (DO-1), PARP, Surviving, Caspase-7, caspase-8, caspase-9, and phosphor-JNK antibodies were purchased from Santa Cruz; phospho-p53 (Ser15) antibody was purchased from US Biological. The level of actin (Sigma-Aldrich) expression served as internal control of protein loading.

### 2.6. Data Statistics

Data of inhibitors' effects are presented as means ± S.E.M., and statistical comparisons were carried out using Student's *t*-test. The calculated probability (*P* value) of 0.05 or less was considered statistically significant.

## 3. Results

### 3.1. LPRP Limits Cellular Expansion in Cultured Cancer Cells

**Figure 1:**
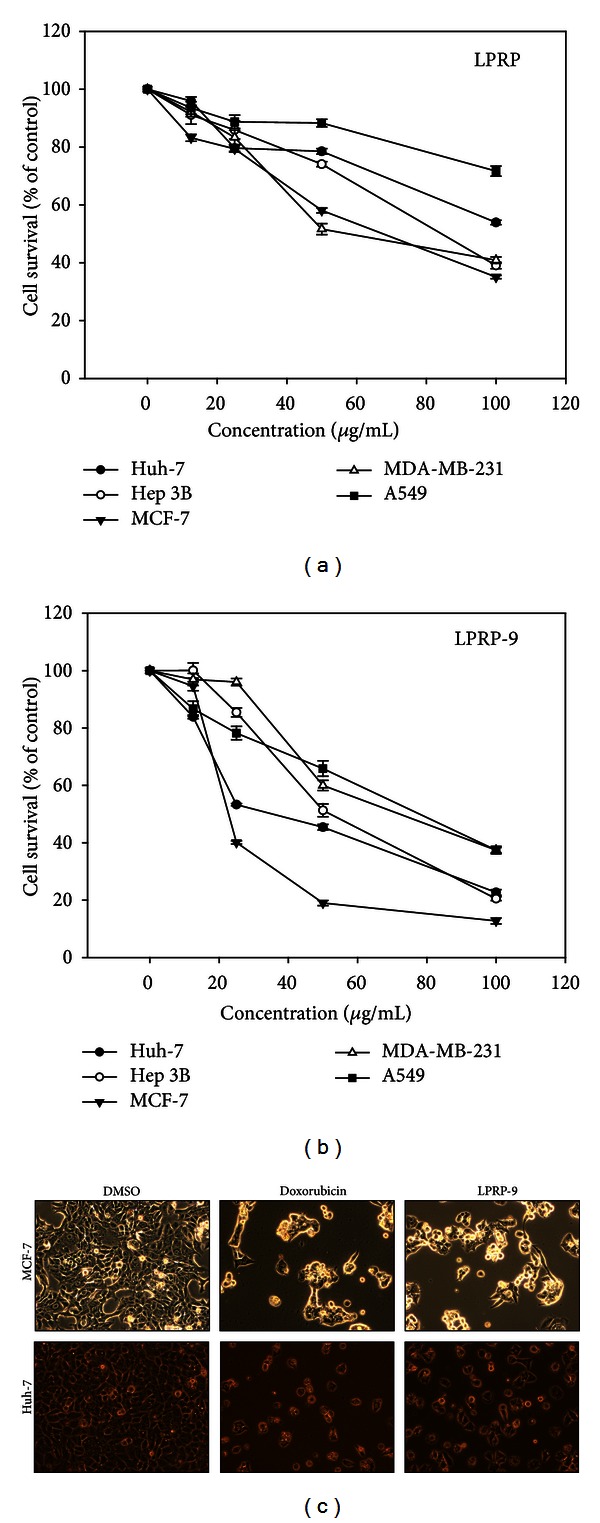
Antiproliferation effects of LPRP on cancer cell lines. Huh-7, Hep 3B, MCF-7, MDA-MB-231, and A549 cells were treated with different indicated concentrations of LPRP (a) or LPRP-9 (b) for 48 h at 37°C in an atmosphere of 5% CO_2_, and the cell survival was measured by MTT assay. (c) MCF-7 and Huh-7 were treated with 5 *μ*M doxorubicin or 20 *μ*g/mL LPRP-9 for 24 h and imaged the cell morphology under a phase contrast inverted microscope at 100x magnification (Axiovert 40; Zeiss).

In order to examine the potential anticancer effect of LPRP, we applied methanol crude extract of LPRP to 5 highly replicating human cancer cell cultures consisting of liver cancer cell lines Huh-7 and Hep 3B, breast cancer cell lines MCF-7 and MDA-MB231, and lung cancer cell line A549. The dose-dependent effect of cell growth and the replicated data are represented as line graphs (Figure 1(a)). The calculated half-maximal-inhibitory concentrations (IC_50_ value) of LPRP were higher than 100 *μ*g/mL for A549 and Huh-7 cells and 84.3  ±  1.0, 67.6  ±  0.8, and 57.6  ±  1.8 *μ*g/mL for Hep 3B, MCF-7, and MDA-MB-231 cells, respectively. The extract was subsequently fractionated by 70% ethanol [11], and selected fractions were tested for their effects on cytotoxicity in the above 5 cell lines again. The effect of the 9th fraction of LPRP, named LPRP-9, was graphed in Figure 1(b), and the calculated IC_50_ values for A549, MDA-MB-231, Hep 3B, Huh-7, and MCF-7 cells were 77.9 ± 1.9, 72.2 ± 1.4, 52.1 ± 2.2, 36.0 ± 0.2, and 23.1 ± 1.3 *μ*g/mL, respectively. Of which, MCF-7 and Huh-7 cells showed more sensitivity to LPRP-9 and are chosen for this study. Under cytohistological observation, treatment of MCF-7 and Huh-7 cells with 20 *μ*g/mL of LPRP-9 produced comparable inhibitory effect to 5 *μ*M of the positive control doxorubicin on day 2 of drug incubation (Figure 1(c)). This study demonstrates that the LPRP-9 fraction possess anticancer property potent enough to warrant consideration as either an adjuvant to cancer chemotherapy or as raw material for extraction of compound(s) for such purposes.

### 3.2. LPRP-9 Regulates the PI3 K and MAPK Pathways in Cancer Cells

**Figure 2:**
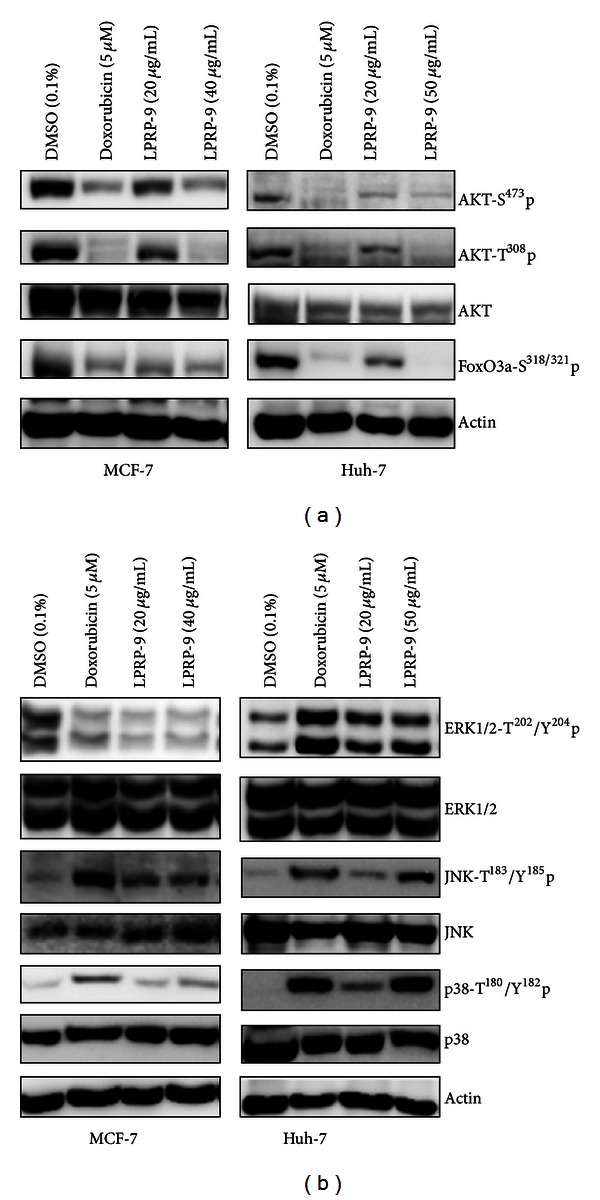
Effects of LPRP-9 on PI3 K/AKT/FaxO and MAPK pathways. Cells were treated with indicated doses of doxorubicin or LPRP-9 for 24 h. (a) Immunoblots results showing AKT activation status by detecting phosphorylation of AKT Thr^308^, Ser^473^, and FaxO3a Ser^318/321^ in MCF-7 (left) and Huh-7 (right) cells. (b) Immunoblots results showing MAPK activation status by detecting phosphorylation of ERK1/2 Thr^202^/Tyr^204^, JNK Thr^183^/Tyr^185^, and p38*α*/*β* Thr^180^/Tyr^182^ in MCF-7 (left) and Huh-7 (right) cells.

To assess the effects of LPRP-9 on cancer growth, we determined the phosphorylation status of principal cell growth pathway downstream effectors, AKT, ERK1/2, p38, and JNK, for the cancer cell lines after 24 hours of exposure to LPRP-9. In both MCF-7 and Huh-7 cells, phosphorylation of AKT Ser^473^ and Thr^308^, normally mediated by mTORC2 and PI3 K/PDK1, respectively [13], was decreased by addition of LPRP-9 in a dose-dependent fashion. This suggests that these upstream signals were blocked (Figure 2(a)). We also observed a reduction in the inhibitory phosphorylation of AKT downstream target forkhead transcription factor FoxO3a, indicating an increase in FoxO3a transcriptional activation possibly via LPRP-9-induced dephosphorylation of FoxO3a Ser^318/321^. Since FoxO3a is also a mediated target of ERK1/2, a component of Ras-MAPK oncogenic pathway [14], we also investigated the MAPK pathway in our project. Our results showed that both LPRP-9 and doxorubicin decreased Thr^202^/Tyr^204^ phosphorylation of ERK1/2 in MCF-7 cells, while perhaps surprisingly, the opposite was true for Huh-7 cells. As for stress-induced MAPK activation, both LPRP-9 and doxorubicin increased JNK Thr^183^/Tyr^185^ and p38 Thr^180^/Tyr^182^ phosphorylation in MCF-7 and Huh-7 cells (Figure 2(b)). These data provide valuable clues hinting that LPRP-9 limits cell growth and promotes cell death in cancer cells.

### 3.3. LPRP-9 Regulates Antideath and Prodeath Factor Expression in Cancer Cells

**Figure 3:**
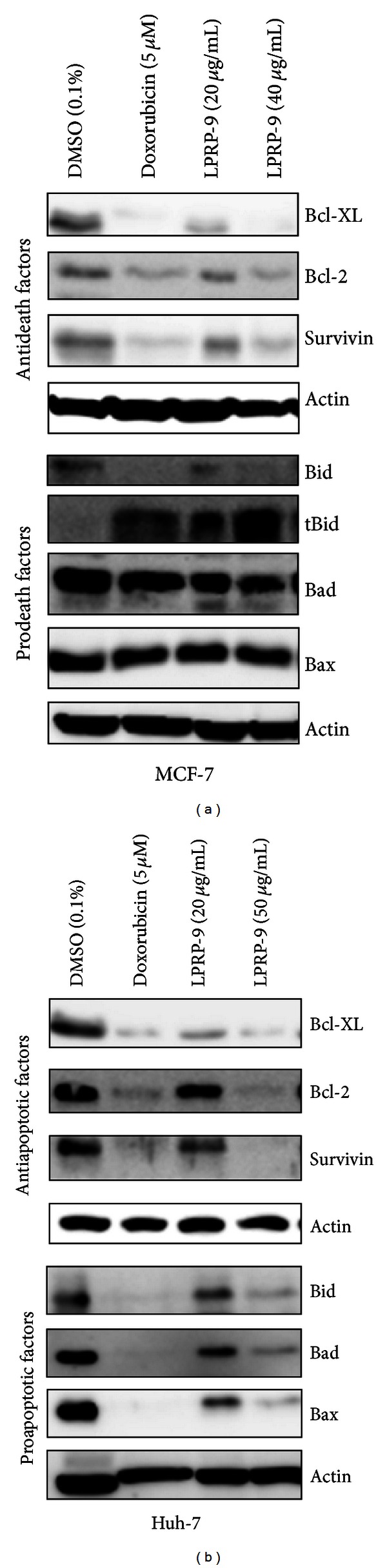
Effects of LPRP-9 on pro- and antideath factors expression. (a) MCF-7 and (b) Huh-7 cells were treated with indicated doses of doxorubicin or LPRP-9 for 24 h. Immunoblots results showing death-associated factors expression by detecting levels of Bcl-XL, Bcl-2, and survivin for antideath factors (upper) and Bid, Bad, and Bax for prodeath factors. The active/truncated form of Bid (tBid) was also detected in MCF-7 cells.

Cellular stress and chemotherapeutics often induce mitochondria-mediated intrinsic death pathway causing cell lethality [15]. To determine factors contributing to LPRP-9-induced cell death, we analyzed the expression of antiapoptotic regulators Bcl-2 and Bcl-XL of the Bcl-2 family and survivin of the inhibitor of apoptosis (IAP) family. Prodeath regulators Bid, Bad, and Bax of the Bax superfamily were also evaluated. In MCF-7 cells, we found that LPRP-9 decreases expression of antideath factors Bcl-2, Bcl-XL, and survivin, while increasing cleavage of Prodeath regulator Bid to the active form tBid, in a dose-dependent fashion. No effects on Bad and Bax expression were observed (Figure 3(a)). In Huh-7 cells, both antideath and Prodeath factors tested were decreased by LPRP-9 in a dose-dependent fashion (Figure 3(b)). This demonstrates that LPRP-9 seems to tip the balance towards cell death in MCF-7 cells, while in Huh-7 cells such effect is undermined.

### 3.4. Activation of Apoptosis and Autophagy Markers in LPRP-9-Treated Cells

**Figure 4:**
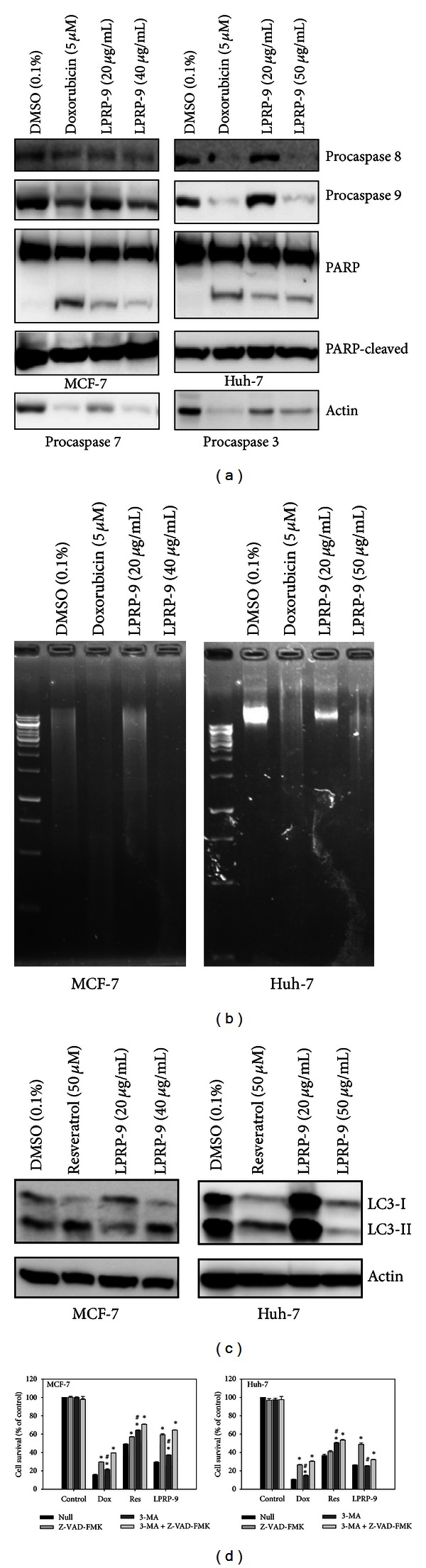
Characterization of apoptotic and autophagic death induced by LPRP-9. (a) Immunoblots results showing indicated doses of LPRP-9- or doxorubicin-induced apoptosis by detecting levels of procaspase 8, procaspase 9, and PARP cleavage for MCF-7 (left) and Huh-7 (right), and procaspase 7 or procaspase 3 for MCF-7 or Huh-7 cells, respectively. (b) Chromosomal fragmentation detection results for MCF-7 (left) and Huh-7 (right) showing chromosome integrity change due to LPRP-9 or doxorubicin treatment. (c) Immunoblots result of autophagy marker LC3 conversion was detected in MCF-7 (left) and Huh-7 (right). (d) Effects of Z-VAD-FMK and 3-MA on doxorubicin, resveratrol or LPRP-9-induced cell death by detecting cell viability using MTT assay at 24 h of drugs incubation. *N* = 3. *P < 0.05 compared with null control within group. ^#^ *P* < 0.05 compared with the Z-VAD-FMK treatment within group.

To characterize LPRP-9-induced cell death, we assayed apoptotic and autophagic markers such as the caspases and LC3 in LPRP-9-treated cancer cells. Doxorubicin and resveratrol are used as positive controls for apoptosis and autophagy, respectively. When treated with high dose of LPRP-9 (40 and 50 *μ*g/mL for MCF-7 and Huh-7 cells, resp., because there is a difference of roughly 10 *μ*g/mL between their IC_50_ values of cytotoxicity), both cell types exhibit increased activation of caspase-8 and caspase-9 as well as increased DNA fragmentation in comparable levels to the positive control doxorubicin after 24 hours of drug incubation (Figures 4(a) and 4(b)). Since two major apoptotic pathways converge on activation of effectors caspase-3 and caspase-7, we also assayed activity of caspase-3 and caspase-7 by cleavage status of PARP, a nuclear substrate of caspase-3 and caspase-7. We found that low dose of LPRP-9 (20 *μ*g/mL for both cell types) was sufficient to cleave PARP in both cancer cells, suggesting that LPRP-9 is capable of inducing apoptosis through activation of caspase cascades even at low doses.

Another critical process for programmed cell death is autophagy. During this process LC3-II, a reliable autophagy marker, is converted from the cytoplasmic form LC3-I to the membrane form LC3-II during autophagy [16]. We found that high dose of LPRP-9 increased LC3-I conversion to LC3-II in MCF-7 cells as much as the positive control resveratrol (50 *μ*M). Interestingly, the same experimental condition caused an overall decrease in LC3 protein expression in Huh-7 cells with no observed increase in LC3-I to LC3-II conversion (Figure 4(c)). To further confirm the pathways involved in LPRP-9-induced cells death, we used inhibitors of apoptosis and autophagy to test their effect on cell survival after LPRP-9 treatment. In our experiments, both Z-VAD-FMK (pan-caspase inhibitor) and 3-methyladenine (3-MA; autophagy inhibitor) protected the two cell lines tested from doxorubicin- or resveratrol-induced cells death to varying degrees. Z-VAD-FMK offers significantly more protection than 3-MA for doxorubicin-treated cells, while 3-MA is more effective for resveratrol-treated cells, indicating that doxorubicin mainly induces apoptosis and resveratrol mainly induces autophagy in the cells tested. Z-VAD-FMK also offers much more protective effect from LPRP-9 for both cells types, while 3-MA offers some protection from LPRP-9 for MCF-7, but none for Huh-7 cells (Figure 4(d)), indicating that the mechanism for LPRP-9-induced cell death is cancer type dependent.

### 3.5. Involvement of P53 in LPRP-9-Induced Cell Death

**Figure 5:**
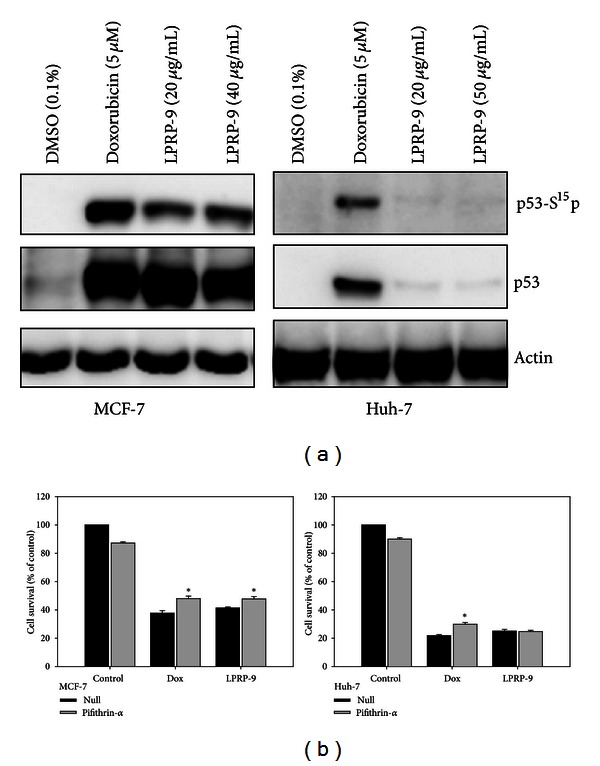
Involvement of p53 in LPRP-9-induced cell death. MCF-7 and Huh-7 were treated with indicated doses of doxorubicin or LPRP-9 for 24 h. (a) Immunoblots results showing p53 activation by detection levels of phosphor-p53 Ser^15^ and p53 protein. (b) Effects of pifithrin-*α* on doxorubicin or LPRP-9 induced-cell death by detecting cell viability using MTT assay at 24 h of drugs incubation. *N* = 3. *P < 0.05 compared with null control within group.

P53 is a tumor suppressor protein that suppresses cell growth either by blocking cell cycle or triggering programmed cell death, primarily via its transcriptional activity for specific gene targets [17]. P53 also plays a major role in cellular response to DNA damage. As p53 Ser^15^ phosphorylation status is reflective of p53 stability and activity, we assayed p53 Ser^15^ phosphorylation to identify p53's involvement in LPRP-9-induced DNA breakage (Figure 4(b)). The results showed that low dose of LPRP-9 induced major increase in p53 Ser^15^ phosphorylation and p53 protein stabilization on par with doxorubicin in p53 wild-type MCF-7 cells, while having minimal effect in p53 mutated Huh-7 cells (Figure 5(a)). We then tested the effect of pifithrin-*α*, a p53 inhibitor, on LPRP-9-induced cell death. Inhibition of p53 transcriptional activity by pifithrin-*α* offered some protection from doxorubicin- and LPRP-9-induced cells death in MCF-7 cells, but had no observed effect on LPRP-9 treated, p53 mutated Huh-7 cells (Figure 5(b)). These results indicate that functional p53 protein is a positive factor that regulates LPRP-9-induced cell death.

### 3.6. AKT Inhibition by a Pure Component Identified from LPRP-9

**Figure 6:**
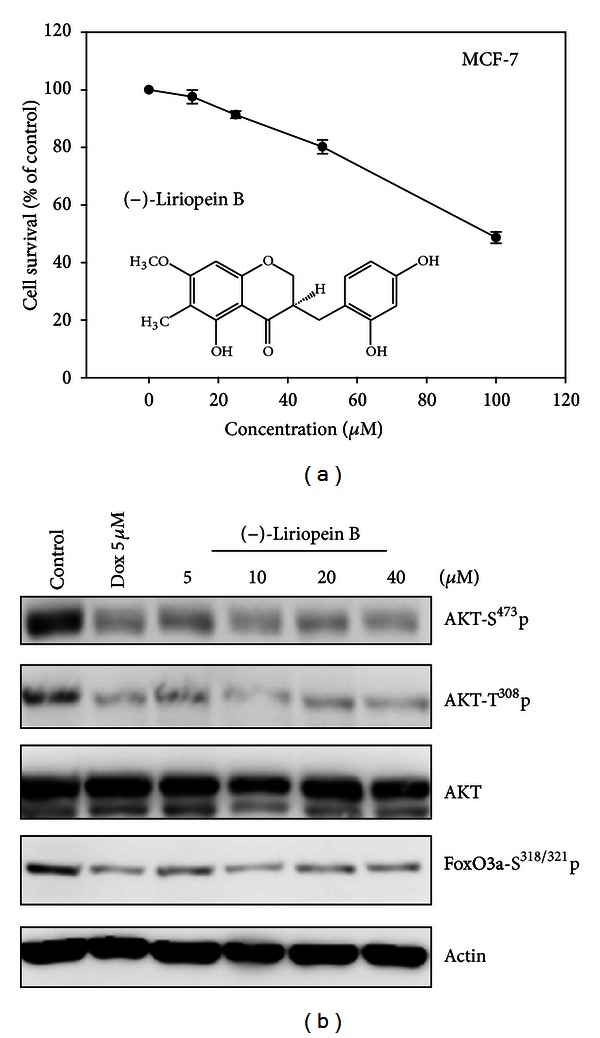
(−)-Liriopein B identified from LPRP-9 inhibits AKT activation. (a) Effect of (−)-Liriopein B on MCF-7 cell growth was measured by MTT assay, which was performed at 48 h after cells exposure to the indicated doses of (−)-Liriopein B. (b) Immunoblots results showing the effect of (−)-Liriopein B on AKT signaling pathway expression by detecting phosphorylation of AKT Thr^308^, Ser^473^, and FaxO3a Ser^318/321^ in MCF-7 cells treated with drugs at the indicated doses for 24 h.

(−)-Liriopein B is a novel compound purified and identified from LPRP-9 and accounts for 1.05% by weight in LPRP-9 [11]. We tested the toxicity effect of (−)-Liriopein B on MCF-7 cells. (−)-Liriopein B inhibits cell survival in a dose-dependent fashion, but the calculated IC_50_ value was 97.9 ± 2.01*μ*M showing moderate toxicity (Figure 6(a)). We inferred that (−)-Liriopein B may disrupt cellular function by inhibiting cell growth instead of inducing cell death. To prove that, we examined the effect of (−)-Liriopein B on PI3K/AKT activity, which was inhibited by LPRP-9, the source of (−)-Liriopein B. We found that (−)-Liriopein B results in AKT Ser^473^ and Thr^308^ dephosphorylation even at the lowest dose tested of 5 *μ*M and also causes dephosphorylation of the AKT downstream target FoxO3a Ser^318/321^ (Figure 6(b)). These results demonstrate that (−)-Liriopein B is at least a component contributing to anticancer activity of LPRP-9 and that it can inactivate PI3K/AKT and activate FoxO3a at a dose far less than the toxic dosage.

## 4. Discussion

The anticancer effect of tuber extract of *Liriope platyphylla* has been reported by one investigation more than ten years ago [18]. The effective dose of the reported *n*-butanol *Lirope* tuber extract, however, is close to 500 *μ*g/mL—a very high concentration which prevents its effective utilization in cancer therapy. It is noteworthy that spicatoside A isolated from the *n*-butanol extracts has activities of anticancer [18] and neuritogenesis [19]. In this study, the methanol extracts of LPRP displayed effect much more potent than the *n*-butanol extract with varying IC_50_ value of antiproliferation effect on several cancer types. We also discovered that the 9th fraction of the 70% methanol extract is especially effective in controlling growth of MCF-7 and Huh-7 cells (Figure 1). Our study stands as the first report to explore actions of LPRP against cancer on the molecular level. On top of this, *Liriope platyphylla* is also a nontoxic food crop that possesses many different medicinal properties and well suited for landscape gardening. Thus, we are suggesting *Liriope platyphylla* as an economically important plant. 

We demonstrated that LPRP-9-induced cell death was mediated by the apoptosis process, which may in turn be regulated by PI3K/AKT or/and MAPK signaling (Figure 4). In contrast to observations in MCF-7cells, LPRP-9 elevated the proliferative signal ERK1/2 and degraded the death factors Bad and Bax in Huh-7 (Figure 2), creating a cellular environment unfavorable for cell death in situations of general cell stress. This might be the underlying reason why Huh-7 cells are more resistant to LPRP-9 treatment than MCF-7 cells. Moreover, Huh-7 cells express the Y220C mutant form of p53 which results in p53 protein surface cavity and decreases its thermal stability [20]. We speculate that it is likely that more than 80% of p53 protein in Huh-7 is unstable under the 37°C culturing condition, accounting for the observed reduction in response of Huh-7 to LPRP-9. LPRP-9 induced a great increase in p53 expression in MCF-7 cells as much as the putative DNA damaging agent doxorubicin, but neither were very effective in Huh-7 cells, indicating different p53 efficacy in these two cells (Figure 5). The involvement of functional p53 in LPRP-induced cell death was also supported by evidence of p53 inhibitor protecting MCF-7 cells from LPRP-9-induced cytotoxicity but has no effect in Huh-7 cells (Figure 5(b)). Even with the lack of functional p53, LPRP-9 being still effective to Huh-7 cells suggests that LPRP-9 acts on cancers via a multitargeting effect which is yet to be elucidated. The multitargeting effect is a concept that brings to light the advantage of botanical crude drug in treatment of diseases. In the case of cancer treatment, modern pharmaceutical attempts to overcome tumor usually consist of targeting single gene or pathway found to be along a critical pathway to malignant development. However, cancer is usually a result of dysfunction of multiple genes and pathways. The desired results may not necessarily occur even if the target is successfully altered [21].

In order to distinguish death pathways induced by LPRP-9 exposures, we applied two well characterized anticancer drugs, doxorubicin and resveratrol, as positive controls. In our experiments, resveratrol induced LC3-II conversion in both tested cells, and 3-MA has a stronger protection effect than Z-VAD-FMK when resveratrol was used, indicating that resveratrol induced mainly autophagic cell death while inducing apoptotic cell death to a lesser degree. In contrast, when incubated with doxorubicin, Z-VAD-FMK showed greater protective effect than 3-MA in both cells tested, indicating that doxorubicin induces mainly apoptotic cell death and to a lesser degree autophagic cell death (Figure 4(d)). With this knowledge, it is interesting to note that Z-VAD-FMK is very effective in protecting both cell lines from cell death by LPRP-9, whereas 3-MA is only marginally effective in protecting MCF-7 cells, indicating that LPRP-9 toxicity is mainly mediated by the apoptotic process. In contrast, 3-MA has no observable effect on LPRP-9 toxicity but eliminated the protective effect of Z-VAD-FMK to LPRP-9-induced cell death in Huh-7 cells. Endogenous levels of LC3 protein are high in Huh-7 cells, which is consistent with previous findings that high expression of LC3 in gastrointestinal tumor benefits cancer development [22]. The resulting LC3 expression pattern correlated with the observed difference in 3-MA effect on resveratrol- and LPRP-9-treated Huh-7 cells, where 3-MA has no observable effect on LPRP-9-treated Huh-7 cells (Figure 4(d)). Elimination of LC3 promotes LPRP-9 toxicity and further supports the protective role of LC3 protein in Huh-7 cells. The lack of effect of 3-MA may be explainable by the different p53 expression and morphology profile of Huh-7 cells.

Since LPRP-9 is a mixture, all of the observations are integrated consequence from all constituents of LPRP-9, and the real gene target of a crude extract is hard to define. Even though we do not yet have comprehensive knowledge of the individual chemical constituents of *Liriope *effective on cancers, we have successfully isolated an extract, LPRP-9, which is effective against two kinds of cancer cell lines. On the molecular level, we have a broad grasp of LPRP-9's range of effect and have identified several markers which will be helpful in identifying a pure compound that will be effective on specific gene targets. As a matter of fact, data gathered from this project, namely the discovery that LPRP-9 regulates PI3 K and MAPK signaling in the two tested cell lines, led us to the identification and testing of the compound (−)-Liriopein B and the validation of PI3K pathway regulation by (−)-Liriopein B in MCF-7 cells (Figure 6). Future studies will guide us further along the path to the discovery of the specific gene target for (−)-Liriopein B. Furthermore, what we learnt from LPRP-9 will also become the research basis for discovering *Liriope platyphylla*'s use in cancer treatment.
